# Uncritical Patriotism and Belief in COVID-19 Conspiracies

**DOI:** 10.3389/fsoc.2022.777650

**Published:** 2022-04-27

**Authors:** Marc Oliver Rieger

**Affiliations:** Department IV – Business Administration, University of Trier, Research Cluster “Cultures in Transition”, Trier, Germany

**Keywords:** SARS-CoV-2, COVID-19, conspiracy theories, conspiracy myths, uncritical patriotism, nationalism

## Abstract

The outbreak of the COVID-19 pandemic has also led to many conspiracy theories. While the origin of the pandemic in China led some, including former US president Donald Trump, to dub the pathogen “Chinese virus” and to support anti-Chinese conspiracy narratives, it caused Chinese state officials to openly support anti-US conspiracy theories about the “true” origin of the virus. In this article, we study whether nationalism, or more precisely uncritical patriotism, is related to belief in conspiracy theories among normal people. We hypothesize based on group identity theory and motivated reasoning that for the particular case of conspiracy theories related to the origin of COVID-19, such a relation should be stronger for Chinese than for Germans. To test this hypothesis, we use survey data from Germany and China, including data from the Chinese community in Germany. We also look at relations to other factors, in particular media consumption and xenophobia.

## 1. Introduction

The COVID-19 pandemic is a global problem. The virus does not discriminate and the fight against it requires international collaboration. It did not take long, however, for the pandemic to become infected with nationalism: while the outbreak of the new disease was officially reported in Wuhan, China on 31 December 2019, just 2 days later the first conspiracy theories circulated in China stipulating that the virus had been developed by the US to harm China (Molter and Webster, 2020; Rieger, 2020a). Later, this escalated with former US president Trump naming the pathogen the “Chinese virus”, and the Chinese government starting a coordinated campaign to question the origin of the virus in China (Davey, 2020; Jaworsky and Qiaoan, 2020; Verma, 2020). As a sidenote, the conflict also reached academia with a letter by three researchers from China to *Nature Human Behavior* demanding that academic papers should not state Wuhan or China as origin of the virus (Zeng et al., 2020).

While from a scientific point of view, the virus' origin is of no relevance for the fight against COVID-19, the political dimension of the conflict is obvious (Jaworsky and Qiaoan, 2020). Additionally, it seems natural that nationalists of all countries, when faced with a disastrous situation in their own country caused by the pandemic, will look for scapegoats elsewhere: uncritical patriotism, the close cousin of nationalism, denies anything bad happening in one's own country (unless, of course, it is caused by other countries or national groups). For Chinese nationalists it is therefore pivotal to deny the origin of the disease in China, while for, e.g., US nationalists it is pivotal to blame China for the hundreds of thousands of deaths in the US. Conspiracy theories play an essential role for both groups, as they conveniently focus responsibility onto an outside group.

The socio-psychological reasons for the formation of uncritical patriotism (as opposed to constructive patriotism, Schatz et al., 1999) and its effect on the belief in “convenient” lies and conspiracy theories have been studied in more depth in recent years, and it has been suggested that focussing on national and ethnic identity rather than on belief in political ideologies can improve our understanding of political phenomena, as suggested. e.g., by Fukuyama (2018). Group identity in itself is not without use for individuals and society (Haidt et al., 2008). It can, however, also have negative effects, and we will focus on one of them here, where the theoretical mechanism can be summarized as follows: group criticism, in particular from outsiders or in times of a perceived threat, can be considered as destructive by members of an identity group (Hornsey and Imani, 2004; Nincic and Ramos, 2012; Adelman and Dasgupta, 2019). Denying criticism can be a solution to the resulting cognitive dissonance. However, it requires certain strategies that can be used to ultimately undermine the relevance of the critique:

- The motivation of the critics can be questioned, as criticism which is not considered to be “caring” usually produces more negative reactions (Hornsey and Imani, 2004).
- The credibility of the critique can be questioned. If a motivation for disbelief (or belief) in a theory is strong, motivated reasoning can provide the required “evidence”.

This connection between group identity, group criticism and motivated beliefs, which may lead to conspiracy beliefs, has been suggested in the case of the beliefs about the “Wuhan Diary” (Fang and Zeller, 2020) held by Chinese in Germany (Wang and Rieger, 2022) and summarizes the theoretical framework for our study exploring the connection between uncritical patriotism (as a particularly strong form of group identity) and conspiracy beliefs. This connection is not unique to COVID-19. Antisemitic conspiracy theories are a prominent example with a long history that, in medieval times, was also at times associated with the outbreak of epidemics (Cohn, 2012). The COVID-19 pandemic, however, gives a topical importance to the connection between uncritical patriotism and conspiracy theories: it has a multitude of consequences for international politics, but also for the potential life or death of millions of people as there is a strong connection between belief in conspiracy theories, protective individual behavior and policies that help to contain the spread of the disease (Allington et al., 2020; Imhoff and Lamberty, 2020; Rieger, 2020a,b; Hornik et al., 2021). This “national” dimension of conspiracy theories has been stronger than in previous pandemics, although rumors and conspiracy theories were of course spread then as well. See, e.g., the in-depth analysis on SARS by Zhou (2003), Ma (2005), and Ma (2008).

Conspiracy theories about COVID-19 with nationalistic undertones, however, did not of course only circulate among Chinese people. In our article, we will also study such beliefs among Germans, in particular the belief that COVID-19 was manufactured in a Chinese laboratory. While such a theory can easily connect with sinophobic stereotypes or at least a perception of Chinese as “out-group” (see Stein et al., 2019 for details and further references), an important ingredient for the thriving of conspiracy theories is lacking: the external criticism of the identity group. Germany is not accused of being the maker or at least the origin of COVID-19, thus national identity does not provide a motivation to reason against the most likely scientific theory of a natural origin of COVID-19 in China. Even though the perceived threat for the identity group due to the virus was surely as severe as in China, we therefore do not expect that uncritical patriotism will lead to a particular increase of belief in conspiracy theories that blame China for the pandemic. Thus, we hypothesize an asymmetry in the effect of uncritical patriotism on conspiracy beliefs: while for Chinese we expect to see a positive relation between uncritical patriotism and belief in anti-China conspiracies, we do not expect to see such a relation for Germans^1^.

In this article, we will verify this hypothesis empirically. Moreover, we will distinguish between the impact of different types of patriotism, in particular uncritical patriotism^2^, the variety closest to nationalism, and we will study some of the correlates, e.g., the effect of COVID-19 on xenophobia, and the potential transmission through social media consumption.

A particularly interesting group of respondents in our survey are Chinese in Germany (more precisely: people with Chinese origin living in Germany). They are “boundary spanners” with plural identities (Carlson, 2009) and can help to disentangle effects of different media and cultural environment from the effects of group identity that are central to our study.

This article is structured as follows: in Section 2, we will describe our surveys, their items and subject pools. In Section 3, we analyze the relation between uncritical patriotism and belief in conspiracy theories. Section 4 summarizes the findings and discusses their limitations as well as future avenues of research.

## 2. Methodology

### 2.1. Sample Characteristics

We use data from an online survey that ran in several waves in Germany, China and among Chinese in Germany^3^ between March 2020 and December 2020 with in total more than 3,000 participants, see Rieger and He-Ulbricht (2020) for a documentation of the first waves. The survey was advertised in several universities. Apart from the main sample from Germany, the survey was also conducted in China in June 2020, and among Chinese in Germany (two waves in summer 2020). Here, the survey was distributed *via* Email and WeChat to a broader community, also outside universities.^4^ In the survey, respondents were asked about a varying number of items regarding COVID-19, in particular their belief in conspiracy theories, but also regarding the origin of COVID-19 and patriotism. The questions were not identical between the waves, thus leading to smaller sample sizes, depending on the interactions studied. Basic demographic characteristics of the sample can be found in Table 1.

**Table 1:** Demographic characteristics of the three samples.

		**German** **(%)**	**China** **(%)**	**Chinese in** **Germany** **(%)**
Age	Average	26	22	33
Gender	Male	38	28	33
	Female	62	72	66
	Other	1	0	1
Highest degree	High school	53	79	9
	Bachelor	29	22	32
	Master or similar	13	0	45
	PhD	2	0	11
	Other	2	0	3
	Pupil	0	0	1
Occupation	Students	81	100	39
	Working	16	0	34
	Others	2	0	26
Time spent in Europe in years (average)				6.0
(median)				3.5
*N*		3,159	135	193

In the following, we describe the items of the survey that are relevant for the following analysis. General questions, e.g., about demographics, are described in Rieger and He-Ulbricht (2020). The data analysis was conducted with SPSS version 25. The data is openly available, see Rieger and He-Ulbricht (2020).

### 2.2. Survey Items: Conspiracy Theories

The conspiracy theory items were elicited in two parts. In the first part, some general statements were presented in random order, mixed with a couple of items on other topics and one about trust in COVID-19 reports in German media and by German institutions:

1. The media try to hide information about the Coronavirus from us.
2. The hype about the Coronavirus was caused by pharmaceutical companies and other groups that benefit from it.
3. The virus is just an excuse for our politicians to trample on our fundamental human rights.

Each item was elicited on a 4-point Likert scale: disagree / somewhat agree / mostly agree / fully agree.

In the second part, more specific conspiracy theories were presented, again in random order, mixed with statements that reflected the scientific consensus (see below):

4. The first patient was an employee of a virus laboratory in Wuhan who got infected by accident.^5^
5. The US Secret Service developed the virus and imported it into Wuhan to damage China.
6. The virus was developed by China at a laboratory for biological weapons and spread due to an accident.
7. The spread of COVID-19 is related to the rollout of 5G networks.
8. Pharmaceutical companies and Bill Gates spread the virus to make money from their patented vaccine.

All items were elicited on a 5-point Likert scale (very unlikely / unlikely / average probability / likely / very likely).

The statements about the scientific consensus were:

9. The virus originated in animals (bats or pangolins) and spread to humans.
10. The virus emanated in Wuhan (China).

We defined scores for conspiracy beliefs as follows, similarly to Rieger (2020a):

- “Neutral conspiracies”: sum of items 1, 2, 3, and 7 (Cronbach's Alpha: 0.761, *N* = 2, 240, minimum theoretical value 4, maximum theoretical value 19, average 5.25).
- Anti-China conspiracies: sum of items 4 and 6 (Cronbach's Alpha: 0.672, *N* = 2, 310, 2 to 10, average 4.23).
- Anti-US conspiracies: sum of items 5 and 8 (Cronbach's Alpha: 0.756, *N* = 2, 308, 2 to 10, average 2.65).

A composite score (“conspiracies score”) was calculated as the sum of these three subscales (Cronbach's Alpha: 0.814, *N* = 2, 234).

The sum of responses to these two questions 9 and 10 was defined as a scale “consensus” (Cronbach's Alpha: 0.564, *N* = 2, 312, possible values between 2 and 10, average 7.97).

In China, the survey was conducted in two waves. In the first wave, we could not ask directly about the conspiracy theories to avoid potential political problems. We therefore instead asked where subjects thought the virus originated: China, USA, elsewhere. For each option we elicited the likelihood on a 4-point Likert scale (yes / rather likely / rather unlikely / no). In the second wave, we did not involve colleagues from China in the data collection and could therefore ask questions more freely. We thus additionally elicited some of the conspiracy theory items as in the other waves.

### 2.3. Survey Items: Patriotism

In some of the waves, we also elicited patriotism and uncritical patriotism. To this end, we used two standard items that have been used frequently in the literature and previous surveys, e.g., the World Value Survey:

- *Patriotism:* I am proud to be German/Chinese.
- *Uncritical patriotism:* We should all fight for our country, whether it is right or wrong.

In both cases, we used (as is standard in the literature) a 4-point Likert scale: disagree completely / mostly disagree / mostly agree / fully agree.

From previous studies, it is known that both variants of patriotism are higher in China than in Germany, which is reflected in our data (see Tables A1, A2).^6^

### 2.4. Survey Items: Media Sources and Other Items

We asked Chinese participants living in Germany about the media sources from which they obtain news. They could select between WeChat, Facebook, Chinese press, German press, German TV, Chinese TV, English language press, and English language TV. Multiple selections were possible.

In fact, WeChat was the most frequently chosen media channel (77%), followed by Chinese press (67%), German press (60%), and German TV (50%). All other channels were chosen 35% or less.

We also elicited among Chinese in Germany how they think that Germans perceive Chinese with the following two items:

1. The Western society is happy to see China decaying.
2. Most Westerners understand the feeling of Chinese people.

These items were elicited on a 4-point Likert scale (Totally disagree / Somewhat disagree / Somewhat agree/ Totally agree).

We computed a score “Perceived anti-Chinese sentiment” as the difference between both items (Cronbach's Alpha: 0.613, *N* = 162).

We also elicited how they think about openly discussing negative issues on the same 4-point Likert scale:

1. I am not in favor of discussing the dark side of society in the public sphere.
2. Without openly discussing negative issues, there is no way to improve the situation.

We defined a score “Hiding dark side” as the difference of both items (Cronbach's Alpha: 0.727, *N* = 143).

Among the German participants, we elicited two items about their attitudes toward Chinese (same 4-point Likert scale):

1. Ultimately, the Chinese are responsible for the Corona pandemic.
2. It would be better if there were fewer Chinese in Germany.

For descriptive statistics on the main variables see Tables A1, A2 in the **Appendix**.

## 3. The Patriotic Side of COVID-19

While relations between patriotism and beliefs about COVID-19 are to be expected among Chinese (for the reasons described in the introduction), there are a priori no strong reasons to expect such effects among Germans.

While there is indeed no relation between (critical) patriotism and conspiracy beliefs, there is a strong positive correlation between uncritical patriotism and all types of conspiracy beliefs (Table 2). These results may suggest that persons who are inclined to uncritical patriotism and persons who believe in conspiracy theories share some common psychological characteristics. The relation confirms previous findings that proponents of far-right political ideologies are more inclined to believe in conspiracy theories than the proponents of centrist political ideologies (van Prooijen et al., 2015), whereby we want to mention for the sake of completeness that van Prooijen et al. (2015) also find that this increased tendency to believe in conspiracy theories also exists among the proponents of far-left political ideologies. The main point here is that the relation between uncritical patriotism and conspiracy beliefs does not distinguish much between conspiracy theories with different contents, confirming our initial hypothesis.

**Table 2:** Correlation between patriotism, uncritical patriotism, and various COVID-19-related beliefs among German students.

**Correlation with**	**Patriotism**	**Uncritical patriotism**
Consensus	0.127*	0
	0.049	0.997
Conspiracies score	−0.016	0.228***
	0.799	<0.001
“Neutral” conspiracies	−0.037	0.193***
	0.571	<0.001
Anti-China conspiracies	0.038	0.173**
	0.561	0.001
Anti-US conspiracies	−0.013	0.213***
	0.84	<0.001
	*N* = 241	*N* = 369

* = significant on the 5% level, ** = 1% level, *** = 0.1% level.

Let us next take a look at the Chinese living in Germany (Table 3). Here, for the anti-US conspiracy theories and also for the neutral conspiracies, the picture is similar (although not statistically significant for the latter ones), but we find a striking difference regarding the anti-China conspiracies: a *negative* correlation between uncritical patriotism and belief in the conspiracy theories. It does not seem surprising that anti-Chinese conspiracies are considered to be anti-patriotic by Chinese and, thus, are considered to be less plausible, but we have to keep in mind that even beliefs in contradicting conspiracy theories are usually positively correlated. This demonstrates that the influence of uncritical patriotism on these beliefs is, indeed, very strong. We observe this effect even more distinctly in the sample of Chinese students: here we find a strong and significant *negative* correlation between patriotism and the belief in the theory that the virus originated in China (Table 4).

**Table 3:** Relationship between uncritical patriotism and belief in various conspiracy theories among Chinese living in Germany.

	**Uncritical patriotism**
Consensus	−0.336**
	0.007
Conspiracies score	0.177
	0.161
“Neutral” conspiracies	0.149
	0.239
Anti-China conspiracies	−0.271*
	0.03
Anti-US conspiracies	0.313*
	0.012
	*N* = 64

* = significant on the 5% level, ** = 1% level.

**Table 4:** Relationship between patriotism and belief about potential origins of COVID-19 among subjects in China.

	**Patriotism**
China	−0.273**
	0.002
USA	0.014
	0.877
Elsewhere	0.044
	0.632
	*N* = 122

** = 1% level.

Finally, we combine the data for Germans and Chinese living in Germany and test the effects of uncritical patriotism and its interaction with nationality on belief in the three dimensions of conspiracy theories (Table 5). At first, for the combined dataset we find that uncritical patriotism is positively related to all types of conspiracy beliefs. This suggests that both the belief in conspiracy theories and uncritical patriotism are related by more fundamental psychological or social characteristics. Chinese, however, are less likely to believe in anti-China theories and are more likely to believe in anti-US theories. As expected, we do not find any nationality effect on the “neutral” conspiracies. Next, we take interaction effects into account (where we consider only the two dimensions with national differences). Due to the small number of Chinese students in our sample, we have to enlarge the sample here by adding non-students. For each population, we compute the individual difference to the average level of uncritical patriotism and denote this difference by “deviation uncritical patriotism”. Then we test the model with the interaction term “Chinese × deviation uncritical patriotism”. It turns out that this factor is strongly significantly negative for anti-China theories, i.e., uncritical patriotism plays a significantly larger role on this for Chinese than for Germans. However, we do not find any significant difference for anti-US theories. It seems that uncritical patriotism does, indeed, lead to a more strongly pronounced rejection of anti-China conspiracy theories, but, at the same time, it does not automatically lead to a higher degree of acceptance of anti-US theories—at least not more so than it does for Germans.

**Table 5:** Effect of uncritical patriotism on belief in conspiracy theories among Germans and Chinese living in Germany.

	**“Neutral” conspiracies**	**Anti-China conspiracies**	**Anti-US conspiracies**	**Anti-China conspiracies**	**Anti-US conspiracies**
Age	0.194***	0.059	0.051	0.087	0.053
	(3.523)	(1.067)	(0.929)	(1.579)	(0.979)
Female	−0.022	0.051	0.087	0.043	0.092*
	(−0.495)	(1.12)	(1.922)	(1.010)	(2.19)
Student				0.009	0.018
				(0.104)	(0.214)
University degree	−0.07	0.038	0.053	0.030	0.008
	(−1.475)	(0.782)	(1.122)	(0.597)	(0.159)
Working	−0.039	−0.122*	−0.096	−0.121	−0.066
	(−0.711)	(−2.219)	(−1.755)	(−1.558)	(−0.853)
Uncritical patriotism	0.20***	0.187***	0.237***		
	(4.458)	(4.122)	(5.288)		
Deviation uncritical				−0.188***	−0.238***
Patriotism				(−4.153)	(−5.287)
Chinese				−0.201***	0.138**
				(−4.283)	(2.967)
Chinese × deviation				0.14**	−0.25
Uncrit. patriotism				(2.995)	(−0.533)
**Sample**	**Students**	**Students**	**Students**	**All**	**All**
*N*	478	480	480	541	541
Adjusted *R*^2^	5.7%	4%	5.8%	7.4%	8.7%

* = significant on the 5% level, ** = 1% level, *** = 0.1% level.

Which other factors can explain the fact that the Chinese living in Germany perceive conspiracy theories about COVID-19 differently than Germans? We tested correlations with the following factors: use of social media (WeChat and Facebook), the idea that one should hide bad things and a perceived anti-Chinese sentiment (all as specified in Section 2). The results are summarized in Table 3: social media consumption increased the belief in anti-US conspiracies, regardless of whether Chinese (WeChat) or American (Facebook) apps were used. More importantly, the idea that one should hide bad things increased the belief in neutral conspiracies and reduced the belief in consensus, but decreased the belief in anti-China conspiracies. The latter effect could probably mean that anti-China conspiracies are considered as talking badly of China which these persons would agree one should not do. A perceived anti-Chinese sentiment increased anti-US conspiracy beliefs and strongly decreased the belief in the scientific consensus, so this perception seems to support all beliefs that are directed against foreigners (as they are seen as opponents).^7^

In conclusion, we find strong evidence for the hypothesis that conspiracy theory beliefs are, on average, more widespread in China, as we expected given the cultural and political differences. Chinese living in Germany are placed between both groups. There are different possible interpretations for these findings: it might be that the difference in media freedom enables the Chinese living in Germany to use more critical thinking, or it could be that cultural adaption or self-selection mechanisms blur the cultural differences in holistic/analytic thinking style. Most likely, it will be a mix of these factors.

The differences between Germans and the Chinese living in Germany, however, become larger when we compare the belief in different types of conspiracy theories: Chinese are generally less likely to believe in anti-China conspiracies and are more likely to believe in anti-US conspiracies than Germans. This difference can be partly explained by (uncritical) patriotism, where for belief in anti-Chinese conspiracies, uncritical patriotism indeed acts differently for Chinese and Germans: it affects only the Chinese. For anti-US conspiracies, we do not find different effects of uncritical patriotism. It just correlates positively with belief in *any* kind of conspiracies (except for anti-China conspiracy theories).

There is further evidence in favor of the hypothesis that the differences regarding the beliefs are self-motivated: agreement with the necessity of “hiding dark sides” decreases anti-China conspiracies, but not anti-US conspiracies, while a perceived anti-China sentiment in Germany (“Westerners are happy to see China decaying”) strongly correlates with anti-US conspiracies and a decreased belief in consensus (but not with other types of conspiracies).

However, we do not find evidence that Chinese media would impact conspiracy beliefs differently than Western media: while the use of WeChat by Chinese in Germany did increase the likelihood of believing in anti-US conspiracies, the use of Facebook did so even more.

We also did not find evidence supporting the idea that the status of Chinese in Germany as a minority increased their overall belief in conspiracy theories, but a larger sample would be needed to answer this question.

All in all, the fact that the virus most probably originated in China is a kind of “inconvenient truth” for many Chinese, even more so if they are very patriotic. This makes it easy for them to believe in conspiracy theories that offer alternative “theories” about the origin of the virus. On the other hand, similar reasons make it more difficult for them to believe in anti-China conspiracy theories.

We also repeated the analysis of Table 4 in Taiwan (*N* = 112 subjects) using the same recruiting method as in China. There—as expected—we did not find a significant relation between uncritical patriotism and the belief in the origin of the virus in the US (ρ = 0.14, *p* = 0.14) nor in the CIA conspiracy theory (ρ = −0.91, *p* = 0.34). The CIA theory was also not widespread: 84.1% found it at least unlikely, only 4.4% found it likely.

This finding again confirms our initial hypothesis: since the Taiwanese as a group did not experience criticism (neither through an origin of the virus in China nor an origin of the virus in the US), they did not feel motivated to reason for any of these conspiracy theories.^8^

## 4. Conclusions

Exceptional events require exceptional explanations: this is a well-known phenomenon that helps to create conspiracy theories whenever world-changing events take place (Leman and Cinnirella, 2007; LeBoeuf and Norton, 2011). COVID-19 was absolutely such a world-changing event, and it is therefore not a surprise that conspiracy theories flourished.

An interesting aspect about COVID-19 is that the pandemic immediately led to nationalist sentiments that were decisive in writing some of the most important conspiracy narratives. In this article, we have studied the interaction between uncritical patriotism on the one hand, and belief in certain types of conspiracy theories on the other hand.

We have seen that in China, conspiracy theories that see the “culprit” outside the national borders, and in particular in the US, are very popular—even among our highly-educated sample. Germans, on the other hand, tend to believe less in conspiracy theories, but if so, they are more likely to believe in anti-Chinese theories. In all samples, we have found a general positive relation between belief in such theories and uncritical patriotism, but basically no relation to (critical) patriotism. However, we have also found that among Chinese, the relation reverts for anti-China conspiracy theories and is strongest for anti-US conspiracies. We again see that uncritical patriotism influences the belief in conspiracy theories in a selective way.

Finally, we want to mention one more finding, namely that in the COVID-19 crisis, sinophobia among Germans correlated significantly with uncritical patriotism, but not with normal (critical) patriotism (Table 6).

**Table 6:** Correlations of the two variants of patriotism with Chinese responsibility and the agreement with the statement that “there are too many Chinese in Germany”.

	**Patriotism**	**Uncritical patriotism**
Chinese responsible for COVID-19	0.175**	0.220**
	(0.003)	(<0.001)
It would be better if there were	0.098	0.179***
fewer Chinese in Germany	(0.96)	(<0.001)
*N*	289	483

Pearson correlation, values in parentheses are p-values. * = significant on the 5% level, ** = 1% level, *** = 0.1% level.

We conclude that conspiracy theories surrounding the origin of COVID-19, fueled by nationalism, seem to have a stable effect on beliefs of a large proportion of Chinese. They shape national differences regarding a historic event that are likely to lead to a permanent difference of its perception between China and Germany. The results for Germany will likely carry over to other Western countries as well, but there is definitely a limitation to our study, as it would have been very interesting to also investigate these effects in the US for two reasons: First, anti-China conspiracies have even been propagated by the former US president, so their impact as well as their relation to uncritical patriotism should be larger. Second, the Chinese population in the US must have been in an even more difficult position in this conflict between anti-US and anti-China conspiracy theories, and it would therefore be very interesting to study their beliefs in more details.

## Data Availability Statement

Publicly available datasets were analyzed in this study. This data can be found at: https://www.uni-trier.de/ universitaet/fachbereiche-faecher/fachbereich-iv/faecher/betrieb swirtschaftslehre/professoren/fin/forschung/research-data.

## Ethics Statement

Ethical review and approval was not required for the study on human participants in accordance with the local legislation and institutional requirements. Written informed consent for participation was not required for this study in accordance with the national legislation and the institutional requirements.

## Author Contributions

The author confirms being the sole contributor of this work and has approved it for publication.

## Funding

This work was supported by the research cluster Cultures in Transition in East Asia and Germany of the University of Trier, funded by the research initiative of the state of Rhineland-Palatinate.

## Conflict of Interest

The author declares that the research was conducted in the absence of any commercial or financial relationships that could be construed as a potential conflict of interest.

## Publisher's Note

All claims expressed in this article are solely those of the authors and do not necessarily represent those of their affiliated organizations, or those of the publisher, the editors and the reviewers. Any product that may be evaluated in this article, or claim that may be made by its manufacturer, is not guaranteed or endorsed by the publisher.

## Footnotes

^1^To be more precise: we do not expect that there is such a relation with the *particular* types of conspiracy theories that are related to nations. We do not exclude the possibility that people who are more prone to uncritical patriotism are—well—more *uncritical*, and thus have a higher belief in *all* types of conspiracy theories. Indeed, we will find this to be the case.

^2^For definitions of different concepts of patriotism and its relations to nationalism, see, e.g., Nincic and Ramos (2012) and Kwak and Matsuda (2014).

^3^More precisely, the sample consisted of people in Germany with Chinese origin, regardless of their nationality. The wave was conducted in Chinese language. For simplicity we refer to them as “Chinese in Germany”. The general sample taken from Germany, however, may also include some respondents of other nationalities, but for simplicity we refer to respondents from this sample simply as “Germans”, similarly for the China sample.

^4^Given the different number of waves, the number of participants between the samples of Germans, Chinese in China, and Chinese in Germany differs accordingly.

^5^This item is not scientifically disproved, but there is also no supporting evidence for it. Omitting it does not lead to significantly different results.

^6^This difference might be explained, e.g., by a cultural difference (individualism vs. collectivism), by the role of institutions, by textbooks and school education, but its explanation is beyond the scope of this article.

^7^We also tested for effects of Chinese versus German or English-language traditional media consumption (TV, newspapers), but did not find any significant effects.

^8^It is important here to note that particularly among young Taiwanese, self-identification as Chinese is nowadays an exception—even among the overall population, only 31.4% identify as Taiwanese *and* Chinese and only 2.7% identify solely as Chinese (NCCU, 2021).

## Appendix

### Descriptive Statistics

**TABLE A1:** Descriptive results for the German sample.

	**1 (%)**	**2 (%)**	**3 (%)**	**4 (%)**		**N**
Uncritical patriotism	4.3	16.1	29.8	49.7		483
Chinese responsible	57	26.6	11.3	5.1		1,671
for COVID-19
Better if there were fewer	95.5	2.8	0.6	1.1		844
Chinese in Germany
	**1 (%)**	**2 (%)**	**3 (%)**	**4 (%)**	**5 (%)**	**N**
Wuhan lab accident narrative	25.7	27.2	30.1	12.9	4.1	2,169
China bioweapon narrative	49.4	28.2	14.9	5.8	1.7	2,167
CIA conspiracy narrative	74	18.4	4.6	2.1	0.8	2168
Bill Gates narrative	85.8	8	3.5	1.7	0.9	2,166
	**2 (%)**	**3 (%)**	**4 (%)**	**5 (%)**	**≥6** **(%)**	**N**
Neutral conspiracies	–	71.2	12.8	5.7	3.7	2,164
Anti-China conspiracies	22.2	16.5	20.7	15.6	13.3	2,165
Anti-US conspiracies	70.9	14.9	6.6	2.8	2.3	2,167

*Higher agreement with the theories/statements is indicated by a higher number. See the main text for details on the survey items*.

**TABLE A2:** Descriptive results in China.

**Origin of virus**	**1 (%)**	**2 (%)**	**3 (%)**	**4 (%)**	**N**	
China	13.9	43.3	40.6	2.1	187	
USA	1.1	24.2	70.4	4.3	186	
Elsewhere	3.3	50.0	43.5	3.3	184	
Uncritical patriotism	15.1	52.8	24.5	7.5	53
Proud of Chinese handling	3.8	5.7	34.0	56.6	53
Dark side to be hidden	13.2	64.2	18.9	3.8	53
Improvement society	3.8	24.5	56.6	15.1	53
	**1 (%)**	**2 (%)**	**3 (%)**	**4 (%)**	**5 (%)**	**N**
Wuhan origin	11.3	32.1	34.0	22.6	0	53
CIA conspiracy narrative	16.5	46.8	24.8	11.0	0	52
Frozen food origin	4	19.2	34.0	29.8	12.8	47

*Higher agreement with the theories/statements is indicated by a higher number. See the main text for details on the survey items*.
